# 15-Deoxy-Δ^12,14^-Prostaglandin J_2_ Upregulates the Expression of LPS-Induced IL-8/CXCL8 mRNA in Vascular Smooth Muscle Cells from Spontaneously Hypertensive Rats

**DOI:** 10.4110/in.2009.9.2.64

**Published:** 2009-04-30

**Authors:** Jung Hae Kim, Hee Sun Kim

**Affiliations:** Department of Microbiology, College of Medicine, and Aging-associated Vascular Disease Research Center, Yeungnam University, Daegu, Korea.

**Keywords:** 15d-PGJ_2_, IL-8/CXCL8, vascular smooth muscle cells

## Abstract

**Background:**

15d-PGJ_2_ has been known to act as an anti-inflammatory agent and has anti-hypertensive effects. As a result of these properties, we examined the effect of 15d-PGJ_2_ on the LPS-induced IL-8/CXCL8 mRNA expression in VSMCs from SHR.

**Methods:**

Effect and action mechanism of 15d-PGJ_2_ on the expression of LPS-induced IL-8/CXCL8 mRNA in VSMCs from SHR and WKY were examined by using real-time polymerase chain reaction, electrophoretic mobility shift assay for NF-κB avtivity, Western blotting analysis for ERK and p38 phosphorylation and flow cytometry for NAD(P)H oxidase activity.

**Results:**

15d-PGJ_2_ decreased the expression of LPS-induced IL-8/CXCL8 mRNA in WKY VSMCs, but increased the expression of LPS-induced IL-8/CXCL8 mRNA in SHR VSMCs. The upregulatory effect of 15d-PGJ_2_ in SHR VSMCs was mediated through PPARγ, and dependent on NF-κB activation and ERK phosphorylation. However, inhibition of the p38 signaling pathway augmented the upregulatory effect of 15d-PGJ_2_ on LPS-induced IL-8/CXCL8 mRNA. A NAD(P)H oxidase inhibitor inhibited the upregulatory effect of 15d-PGJ_2_ on LPS-induced IL-8/CXCL8 mRNA expression in SHR VSMCs, and an increase in NAD(P)H oxidase activity was detected in SHR VSMCs treated with 15d-PGJ_2_/LPS.

**Conclusion:**

Our results indicate that the upregulatory effect of 15d-PGJ_2_ on LPS-induced IL-8/CXCL8 expression in SHR VSMCs is mediated through the PPARγ and ERK pathway, and may be related to NAD(P)H oxidase activity. However, p38 inactivation may also play an important role in 15d-PGJ_2_/LPS-induced IL-8/CXCL8 expression in SHR VSMCs.

## INTRODUCTION

Infiltration of inflammatory cells and oxidative stresses in vascular walls have been shown to contribute to the pathogenesis of hypertension (1-4), and monocytes/macrophages infiltration and the proliferation of VSMCs and endothelial cells in arterial walls are mediated by chemokines (5,6). Chemokine IL-8/CXCL8 is known to play an important role in the migration of monocytes into the subendothelial space in the early phase of atherosclerosis, and along with MCP-1/CCL2, plays an important role in the pathogenesis of atherosclerosis (7). In addition, elevated levels of IL-8/CXCL8 are associated with an increased risk of future coronary artery disease (8). IL-8/CXCL8 may directly enhance membrane permeability to Ca^2+^; thus, inducing vasoconstriction in the smooth muscle cells (9). Moreover, we have previously shown that expression of IL-8/CXCL8 in SHR VSMCs is stronger than in WKY VSMCs (10). Therefore, it has been suggested that IL-8/CXCL8 is also involved in the pathogenesis and maintenance of hypertensive vascular wall formation in hypertension.

PPAR ligands have been known to reduce systemic blood pressure (11-13), increase production of a potent endogenous vasodilator, NO and act as an anti-inflammatory agent (14,15). 15d-PGJ_2_, a metabolite of prostaglandin PGD_2_, is a natural and high affinity ligand for PPARγ (16). It is known to produce anti-hypertensive effects, such as inhibiting cell migration and proliferation in rat and human VSMCs, and stimulating HO-1 expression in rat VSMCs (17-19). 15d-PGJ_2_ also inhibits the production of inflammatory mediators (TNF-α, IL-6, IL-1β, IL-2, IP-10, MCP-1, gelatinase B, and cyclooxygenase-2) and reduces the expression of iNOS (15,20-22). As a result of these effects, 15d-PGJ_2_ has been suggested to be a potential therapeutic compound for use as an anti-inflammatory agent. However, there are also evidences that 15d-PGJ_2_ can promote inflammation (23-26). Thus, the role and effects of 15d-PGJ_2_ on inflammation is complex and remains controversial. Moreover, the precise role of 15d-PGJ_2_ in hypertensive VSMCs is not yet fully understood.

Therefore, the aim of this study is to investigate the action mechanism of 15d-PGJ_2_ on LPS-induced IL-8/CXCL8 expression in VSMCs from SHR.

## MATERIALS AND METHODS

### Reagents

Trizol reagent, lipofectamine 2000 and rat p38 siRNA oligomers were purchased from Invitrogen (Carlsbad, CA). Dulbecco's phosphate-buffered saline (PBS), Dulbecco's modified Eagle's medium (DMEM), penicillin-streptomycin and fetal bovine serum (FBS) were purchased from Gibco/BRL (Life Technologies, Gaithersburg, MD). 15d-PGJ_2_ and GW9662 were purchased from Biomol (Plymouth Meeting, PA). *Escherichia coli* LPS (O111:B4), diphenyleneiodonium chloride (DPI), dithiothreitol (DTT), phenylmethylsulfonyl fluoride (PMSF), pepstatin, leupeptin, autipain, and aprotinin were obtained from Sigma Chemical Co. (St. Louis, MO). MAPK inhibitors, 2'-amino-3'methoxyflavone (PD98059), 4-(4-fluorophenyl)-2-(4-nitrophenyl)-5-(4-pyridyl)-1H-imidazole (PD169316), and NF-κB inhibitor, (E)3-[(4-Methylphenyl)sulfonyl]-2-propenenitrile (Bay 11-7082) were purchased from Calbiochem (San Diego, CA). Dichlorofluorescein diacetate (DCF-DA) was obtained from Molecular probes (Eugene, OR). Nitrocellulose transfer membranes were obtained from Schleicher & Schuell Bioscience (Dassel, Germany). [α-^32^P]dCTP was purchased from Dupont-New England Nuclear (Boston, MA). Oligonucleotide primers for polymerase chain reaction (PCR) of IL-8/CXCL8, PPAR and β-actin were synthesized by Bionics (Seoul, Korea). The LightCycler FastStart DNA SYBR Green I Mix was obtained from Roche (Mannheim, Germany). Anti-NF-κB, Phospho-ERK and phospho-p38 antibodies were obtained from Cell Signaling Technology (Danvers, MA). The γ-tubulin antibody was obtained from Sigma Chemical Co. (St Louis, MO). All other reagents were from pure-grade commercial preparations.

### Experimental animal

Specific pathogen-free male inbred WKY and SHR, 20 to 30 weeks of age, were purchased from Japan SLC Inc. (Shizuoka, Japan). All experimental animals received autoclaved food and bedding to minimize exposure to viral or microbial pathogens. The rats were cared for in accordance with the Guide for the Care and Use of Experimental Animals of Yeungnam Medical Center.

### VSMCs preparation

VSMCs were obtained from the thoracic aortas of 20- to 30-week-old male WKY and SHR as described previously (25). VSMCs were cultured in Dulbecco's modified Eagle's medium (DMEM) that was supplemented with 10% FBS and 1% penicillin-streptomycin. Cells were detached using 0.25% trypsin/EDTA and seeded into 75-cm^2^ tissue culture flasks at a density of 10^5^ cells per ml. All experiments were conducted at cell passage 3 to 7. Prior to stimulation, 95% confluent VSMCs were serum-starved overnight by incubating in DMEM with 0.1% FBS. Cell cultures were incubated in a humidified incubator at 37℃ and 5% CO_2_ in the presence or absence of stimuli for the indicated times.

### Preparation of total RNA and real-time polymerase chain reaction (real-time PCR)

Total RNA was extracted using a Trizol reagent in accordance with the manufacturer's instructions. The quantity of total RNA obtained was determined by measuring optical density (OD) at 260 and 280 nm. Real-time PCR for the amplification of IL-8/CXCL8 and PPARγ in VSMCs was performed using a LightCycler (Roche). RNA was reverse transcribed to cDNA from 1 µg of total RNA, and then subjected to real-time PCR. PCR was performed in triplicate. The total PCR volume was 20 µl and contained the LightCycler FastStart DNA SYBR Green I mix (Roche), appropriate primer and 2 µl of cDNA. Prior to PCR amplification, the mixture was incubated at 95℃ for 10 min, and the amplification step consisted of 45 cycles of denaturation (10 s at 95℃), annealing (5 s at the primer-appropriate temperature), and extension (10 s at 72℃) with fluorescence detection at 72℃ after each cycle. After the final cycle, melting point analyses of all samples were performed over the range of 65 to 95℃ with continuous fluorescence detection. β-actin expression levels were used for sample normalization. Results for each gene were expressed as the relative expression level compared with β-actin. The primers used for PCR were as follows: for IL-8/CXCL8 (365 bp) sense, 5'-gaagatagattgcaccga-3'; antisense, 5'-catagcctctcacacatttc-3', for PPARγ (359 bp): sense, 5'-tgaggagaagtcacactctg-3'; antisense, 5'-tgggtcagctcttgtgaatg-3' and for β-actin (101 bp): sense, 5'-tactgccctggctcctagca-3'; antisense, 5'-tggacagtgaggccaggatag-3'. The level of IL-8/CXCL8 mRNA was determined by comparing experimental levels to the standard curves and was expressed as the fold of relative expression.

### Electrophoretic mobility shift assay (EMSA)

Nuclear extracts were prepared as described previously (25). Cells were washed three times with cold PBS, then scraped and harvested by centrifugation. Cell pellets were resuspended and incubated on ice for 15 min in 400 µl of hypotonic buffer A (10 mmol/l HEPES, 10 mmol/l KCl, 1.5 mmol/l MgCl_2_, 0.5 mmol/l DTT, 0.1 mmol/l PMSF, 10 µg/ml pepstatin, 10 µg/ml leupeptin, 10 µg/ml autipain, and 10 µg/ml aprotinin). Nonidet P-40 was then added to a final concentration of 2.5%, and the cells were vortexed for 10 s. Nuclei were separated from the cytosol by centrifugation at 12,000 g for 15 s. Pellets were resuspended in 40 µl of hypotonic buffer C (20 mmol/l HEPES, 25% glycerol, 0.4 mol/l NaCl, 1 mmol/l EDTA, 1 mmol/l EGTA, 0.5 mmol/l DTT, 0.1 mmol/l PMSF, 10 µg/ml pepstatin, 10 µg/ml leupeptin, 10 µg/ml autipain, and 10 µg/ml aprotinin). Samples were sonicated at level 3-4 for 2-3 s, followed by centrifugation for 10 min at 4℃. The nuclear protein concentration was determined using the Bradford assay (Bio-Rad, Richmond, CA). A consensus sequence for the NF-κB DNA binding site (5'-AGTTGAGGGGACTTTAGGC-3') (*sc*-2505; Santa Cruz Biotechnology, Santa Cruz, CA) was labeled with [α-^32^P]dCTP using a random-primed DNA labeling kit (Roche). The mutant NF-κB binding sequence was identical to *sc*-2505 except for a "G" → "C" substitution in the NF-κB DNA binding motif (*sc*-2511; Santa Cruz Biotechnology). DNA The labeled DNA was purified over an S-200 HR column (Pharmacia, Piscataway, NJ) to remove unbound nucleotides. Nuclear protein extracts were incubated at room temperature for 20 min with approximately 50,000 cpm of labeled oligonucleotides that were suspended in the binding buffer [200 mmol/l HEPES, 500 mmol/l KCl, 10 mmol/l EDTA, 50% glycerol, 10 mmol/l DTT, 1 mg/ml BSA, 1 µg/µl poly (dI-dC)]. Following this incubation, samples were resolved on 4% polyacrylamide gels at 140 V and exposed to film.

### Western blotting

Total lysates were prepared in a PRO-PREP buffer (iNtRON, Seoul, Korea). Protein concentrations were determined by a Bradford assay using bovine serum albumin as a standard. Thirty-micrograms of the protein samples were separated on 10% SDS-polyacrylamide gels, and then transferred to nitrocellulose membranes. The membranes were soaked in 5% nonfat dried milk in TBST (10 mmol/l Tris-HCl pH 7.5, 150 mmol/l NaCl and 0.05% Tween-20) for 1 h and then incubated for 16~18 h with primary antibodies against phospho-ERK, phospho-p38 and γ-tubulin at 4℃. Membranes were washed three times with TBST for 10 min and then incubated with horseradish peroxidase-conjugated secondary antibody for 1 h at 4℃. After incubation with the secondary antibody, the membranes were rinsed three times with TBST for 10 min and antigen-antibody complex was detected using an enhanced chemiluminescence detection system (LAS-3000, Fujifilm, Tokyo, Japan).

### Small interfering RNA

To confirm whether the p38 pathway contributes to the inhibitory effect of 15d-PGJ_2_ on LPS-induced IL-8/CXCL8 expression, p38 expression was silenced using small interfering RNA (siRNA). VSMCs were plated on 24-well plates and grown to 90% confluence. VSMCs were then transfected with p38 siRNA oligomers (20 nmol/l) using lipofectamine 2000 in accordance with the manufacturer's instructions. After 24 h of incubation, VSMCs were placed in growth medium for 24 h before the experiments. Cells were then cultured in the presence or absence of stimuli for 4 h. Sense and antisense oligonucleotides corresponding to the rat p38 siRNA sequence: sense, 5'-uacauuugcgaaguucaucuucggc-3'; antisense, 5'-gccgaagaugaacuucgcaaaugua-3' was purchased from Invitrogen (Carlsbad, CA).

### Flow cytometry for ROS generation

ROS production was measured using flow cytometric analysis of DCF-DA-stained cells. In brief, VSMCs were grown to 70% confluence in serum-enriched DMEM at 37℃ in 5% CO_2_. The medium was then replaced with serum-free DMEM and the cells were cultured in the presence or absence of stimuli for the indicated times. Cells were incubated in the dark with DCF-DA (50 µmol/l) for 1 h at 37℃, scraped, and resuspended in PBS. Fluorescence was monitored using flow cytometry (FACScan, Becton Dickison).

### Statistical analysis

Results are expressed as means±SD from at least three or four independent experiments. For comparison between multiple groups, statistical significance was determined by the Mann-Whitney test using the SPSS version 12.0.

## RESULTS

### Effect of 15d-PGJ_2_ on the LPS-induced IL-8/CXCL8 expression in SHR VSMCs

**Figure 1:**
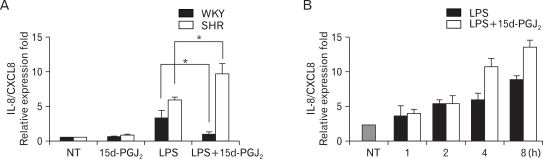
Effect of 15d-PGJ_2_ on the expression of LPS-induced IL-8/CXCL8 mRNA in VSMCs from SHR and WKY, and the time course of 15d-PGJ_2_/LPS-induced IL-8/CXCL8 mRNA expression in SHR VSMCs. (A) VSMCs were untreated (NT) or treated with LPS (1 µg/ml) or/and 15d-PGJ_2_ (10 µM) for 4 h, and the total RNA was analyzed by real-time PCR. Bars represent means±SD from three independent experiments. ^*^p<0.05 vs. VSMCs treated with LPS alone. (B) SHR VSMCs were untreated (NT) or treated with LPS (1 µg/ml) or LPS plus 15d-PGJ_2_ (10 µM) simultaneously (15d-PGJ_2_/LPS) for the indicated times and the total RNA was analyzed by real-time PCR. Bars represent means±SD from three independent experiments.

We examined the differential effect of LPS on IL-8/CXCL8 mRNA expression in SHR VSMCs in comparison to WKY VSMCs. From this experiment, we found that the expression of LPS-induced IL-8/CXCL8 mRNA was greater in SHR VSMCs than WKY. Real-time PCR was performed on VSMCs after they were untreated (NT) or treated with LPS (1 µg/ml), 15d-PGJ_2_ (10 µM) or LPS plus 15d-PGJ_2_ simultaneously (15d-PGJ_2_/LPS) for 4 h. 15d-PGJ_2_ treatment had different effects on SHR VSMCs relative to WKY VSMCs, where 15d-PGJ_2_ had upregulatory effect on LPS-induced IL-8/CXCL8 mRNA expression in SHR VSMCs and suppressive effect on LPS-induced IL-8/CXCL8 mRNA expression in WKY VSMCs. 15d-PGJ_2_ alone did not induce IL-8/CXCL8 mRNA expression in SHR VSMCs significantly (Fig. 1A). The time course of 15d-PGJ_2_/LPS-induced IL-8/CXCL8 mRNA expression was determined in SHR VSMCs over a 0 to 8 h time period. In this experiment, we found that the expression of IL-8/CXCL8 mRNA induced by 15d-PGJ_2_/LPS was almost the same as that for cells treated with LPS alone until 2 h after treatment. However, the expression levels of IL-8/CXCL8 mRNA induced by 15d-PGJ_2_/LPS were significantly greater than those in the cells treated with LPS alone from the 4 h period (Fig. 1B).

### Action mechanisms of 15d-PGJ_2_ on LPS-induced IL-8/CXCL8 expression in SHR VSMCs

**Figure 2:**
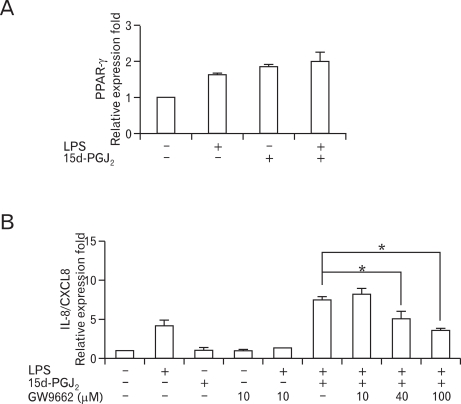
Upregulatory effect of 15d-PGJ_2_ on LPS-induced IL-8/CXCL8 mRNA expression is dependent on the PPARγ pathway in SHR VSMCs. (A) VSMCs were untreated (NT) or treated with LPS (1 µg/ml) and/or 15d-PGJ_2_ (10 µM) for 4 h, and the total RNA was analyzed by real-time PCR. Bars represent means±SD from three independent experiments. (B) VSMCs were untreated or treated with LPS (1 µg/ml) and/or 15d-PGJ_2_ (10 µM) in the absence or presence of GW9662 (10, 40, and 100 µM) for 4 h. Bars represent means±SD from three independent experiments. ^*^p<0.05 vs. VSMCs treated with 15d-PGJ_2_/LPS.

It is widely accepted that 15d-PGJ_2_ exerts its effects on pro-inflammatory genes in cells through PPARγ dependent or PPARγ independent mechanisms (27-29). Before evaluating whether the mechanism of the upregulatory action of 15d-PGJ_2_ in SHR VSMCs was PPARγ-dependent, we determined the expression pattern of PPARγ mRNA in SHR VSMCs treated with 15d-PGJ_2_/LPS. There was no meaningful difference between the level of PPARγ expression induced by 15d-PGJ_2_/LPS and that induced by 15d-PGJ_2_ alone (Fig. 2A). To evaluate whether the upregulatory effect of 15d-PGJ_2_ in SHR VSMCs is mediated by PPARγ, the effect of GW9662, a PPARγ antagonist, was tested in SHR VSMCs. GW9662 blocked LPS-induced IL-8/CXCL8 mRNA expression at the dose of 10 µM. And, although 10 µM of GW9662 did not block the upregulatory effect of 15d-PGJ_2_ on LPS-induced IL-8/CXCL8 mRNA expression, the high doses (40 and 100 µM) of GW9662 inhibited the upregulatory effect of 15d-PGJ_2_ on LPS-induced IL-8/CXCL8 mRNA expression (Fig. 2B).

**Figure 3:**
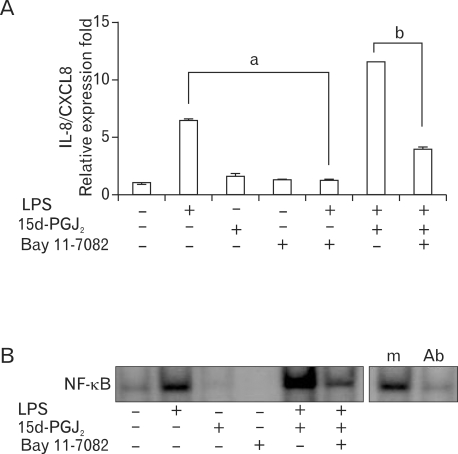
Upregulatory effect of 15d-PGJ_2_ on LPS-induced IL-8/CXCL8 mRNA expression is dependent on NF-κB activation in SHR VSMCs. (A) VSMCs were untreated or treated with LPS (1 µg/ml) and/or 15d-PGJ_2_ (10 µM) in the absence or presence of Bay11-7082 (10 µM) for 4 h. Bars represent means±SD from three independent real-time PCR experiments. a: p<0.05 vs. VSMCs treated with LPS alone. b: p<0.05 vs. VSMCs treated with 15d-PGJ_2_/LPS. (B) Specific binding activity of NF-κB from nuclear extracts was assessed by EMSA. Aliquots of the nuclear extract were incubated with a 100-fold excess of the mutant probe (m) or with 2 µg of the anti NF-κB antibody before EMSA. Data shown are representative of four independent experiments.

To further understand the nature of the upregulatory effect of 15d-PGJ_2_ on LPS-induced IL-8/CXCL8 expressions in SHR VSMCs, the role of NF-κB activation was investigated. Bay11-7082 is known to selectively block the phosphorylation of IκB-α; thus, preventing the activation and nuclear translocation of NF-κB. Real-time PCR and EMSA were performed on VSMCs after they were untreated or treated with LPS (1 µg/ml) and/or 15d-PGJ_2_ (10 µM) in the absence or presence of Bay11-7082 (10 µM) for 4 h. Bay11-7082 remarkably blocked the upregulatory effect of 15d-PGJ_2_ on LPS-induced IL-8/CXCL8 mRNA expression. And it also remarkably blocked LPS-induced IL-8/CXCL8 expression (Fig. 3A). However, in spite of negative NF-κB activity in cells treated 15d-PGJ_2_ alone, NF-κB activity in SHR VSMCs treated with15d-PGJ_2_/LPS was remarkably increased compared to the activity in cells treated with LPS alone (Fig. 3B).

**Figure 4:**
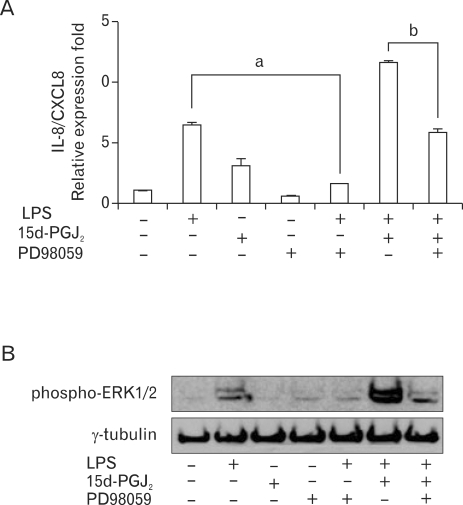
Upregulatory effect of 15d-PGJ_2_ on LPS-induced IL-8/CXCL8 expression is mediated through the ERK pathway in SHR VSMCs. (A) VSMCs were untreated (NT) or pretreated with PD98059 (ERK inhibitor, 10 µM) for 30 min, and then untreated or treated with LPS (1 µg/ml) and/or 15d-PGJ_2_ (10 µM) for 4 h. Real-time PCR was performed after total mRNAs were isolated. Bars represent means±SD from three independent experiments. a: p<0.05 vs. VSMCs treated with LPS alone. b: p<0.05 vs. VSMCs treated with 15d-PGJ_2_/LPS. (B) Cell lysates were separated on 10% SDS-polyacrylamide gels and then immunoblotted with the phospho-ERK antibody. The data shown are representative of three independent experiments.

**Figure 5:**
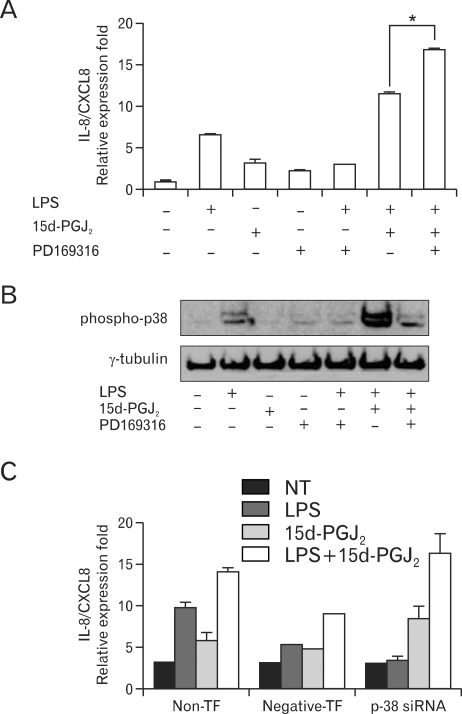
Blocking of p38 phosphorylation increased the expression of 15d-PGJ_2_/LPS-induced IL-8/CXCL8 mRNA expression in SHR VSMCs. (A) VSMCs were untreated (NT) or pretreated with PD169316 (p38 inhibitor, 10 µM) for 30 min, and then untreated or treated with LPS (1 µg/ml) and/or 15d-PGJ_2_ (10 µM) for 4 h. Real-time PCR was performed after total mRNAs were isolated. Bars represent means±SD from three independent experiments. ^*^p<0.05 vs. VSMCs treated with 15d-PGJ_2_/LPS. (B) Cell lysates were separated on 10% SDS-polyacrylamide gels and then immunoblotted with the phospho-p38 antibody. Data shown are representative of four independent experiments. (C) VSMCs were plated on 24-well plates, grown to 90% confluence and then transfected with p38 siRNA oligomers (20 nmol/l). VSMCs were then untreated or treated with LPS (1 µg/ml) and/or 15d-PGJ_2_ (10 µM) for 4 h. Bars represent means±SEM from three independent experiments.

Next we investigated whether the MAPK signaling pathways are involved in the upregulatory effect of 15d-PGJ_2_ in SHR VSMCs. After VSMCs were untreated (NT) or pretreated with PD98059 (ERK inhibitor, 10 µM, 4A), or PD169316 (p38 inhibitor, 10 µM, 5A) for 30 min, cells were untreated or treated with LPS (1 µg/ml) and/or 15d-PGJ_2_ (10 µM) for 4 h. Real-time PCR was then performed on these treated cells. In addition, these results were further confirmed by investigating the phosphorylation of MAP kinases in VSMCs that had been treated with 15d-PGJ_2_/LPS. The expression of 15d-PGJ_2_/LPS-induced IL-8/CXCL8 mRNA was decreased by the ERK inhibitor PD98059. And the expression of LPS alone-induced IL-8/CXCL8 mRNA was also decreased by PD98059 (Fig. 4A). However, although ERK phosphorylation in cells treated 15d-PGJ_2_ alone was not detected, a remarkable increase in ERK phosphorylation in VSMCs that were treated with 15d-PGJ_2_/LPS relative to VSMCs that were treated with LPS alone was also detected (Fig. 4B). PD169316 increased the IL-8/CXCL8 mRNA expression in VSMCs stimulated with 15d-PGJ_2_/LPS, rather than inhibiting IL-8/CXCL8 expression (Fig. 5A). Moreover, 15d-PGJ_2_ decreased LPS-induced p38 phosphorylation (Fig. 5B). More specifically, blocking p38 phosphorylation caused an increase in 15d-PGJ_2_/LPS-induced IL-8/CXCL8 expression. To confirm this result, real-time PCR using p38-directed small interfering RNA (siRNA) was performed. In this experiment we found that while LPS inhibited the expression of IL-8/CXCL8 mRNA, 15d-PGJ_2_ induced IL-8/CXCL8 expression and 15d-PGJ_2_/LPS increased IL-8/CXCL8 mRNA expression in p38 siRNA transfected SHR VSMCs (Fig. 5C).

### Effect of NAD(P)H oxidase activity on 15d-PGJ_2_/LPS-induced IL-8/CXCL8 expression in SHR VSMCs

VSMCs generate reactive oxygen species (ROS), which play an important role in the pathogenesis of hypertensive vascular injury. A major source of ROS is NAD(P)H oxidase. Therefore, we investigated whether NAD(P)H oxidase activity is related to the upregulatory effect of 15d-PGJ_2_ on LPS-induced IL-8/CXCL8 expressions in SHR VSMCs.

**Figure 6:**
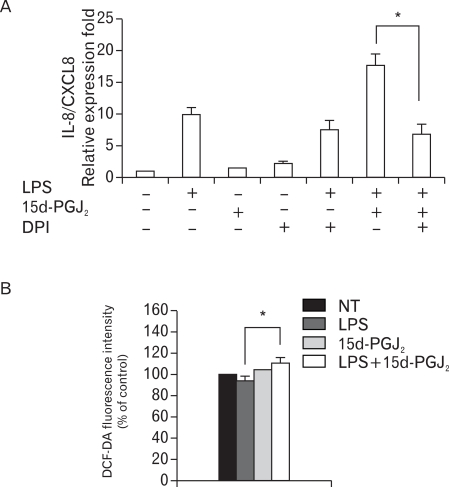
Activity of NAD(P)H oxidase mediates the upregulatory effect of 15d-PGJ_2_ on the expression of LPS-induced IL-8/CXCL8 mRNA in SHR VSMCs. (A) VSMCs were untreated or treated with LPS (1 µg/ml) and/or 15d-PGJ_2_ (10 µM) in the absence or presence of DPI (10 µM) for 4 h. Bars represent means±SD from three independent experiments. ^*^p<0.05 vs. VSMCs treated with 15d-PGJ_2_/LPS. (B) VSMCs were untreated or treated with LPS (1 µg/ml) and/or 15d-PGJ_2_ (10 µM) for 4 h, stained with DCF-DA (50 µM) for ROS detection, and subjected to flow cytometry. Bars represent means±SD from four independent experiments. ^*^p<0.05 vs. VSMCs treated with LPS alone.

Real-time PCR was performed on VSMCs SHR after they were untreated or treated with LPS (1 µg/ml) and/or 15d-PGJ_2_ (10 µM) in the absence or presence of flavin containing oxidase inhibitor, DPI (10 µM) for 4 h. DPI remarkably decreased the expression of 15d-PGJ_2_/LPS-induced IL-8/CXCL8 mRNA; thus, blocking the upregulatory effects of 15d-PGJ_2_ on LPS-induced IL-8/CXCL8 expression (Fig. 6A). To support these results, the ability of 15d-PGJ_2_/LPS to induce NAD(P)H oxidase activity was examined in SHR VSMCs. ROS generation in SHR VSMCs was measured by flow cytometric analysis of DCF-DA-stained VSMCs. DCF-DA fluorescence can be also used as a measurement of NAD(P)H oxidase activity. From this analysis, SHR VSMCs treated with 15d-PGJ_2_/LPS was shown to increase DCF-DA fluorescence slightly compared to those treated with LPS alone (Fig. 6B).

## DISCUSSION

In relation to IL-8/CXCL8 expression, 15d-PGJ_2_ was shown to have pro-inflammatory effects in SHR VSMCs and anti-inflammatory effects in WKY VSMCs. Wakino et al. (30) also observed similar differential effects of PPARγ ligands on vascular tissues from SHR and WKY. They demonstrated that pioglitazone reduces the stimulated Rho-kinase activity in the vascular tissue from SHR, but not WKY. Troglitazone markedly decreased the expression of TGFβ-1, PDGF, or bFGF mRNAs in SHR VSMCs, but not in WKY VSMCs (12). Therefore, these different effects of PPAR ligands on SHR and WKY VSMCs indicate that diverse, complex pathways mediate the action of PPARg ligands on hypertension.

The upregulatory effect of 15d-PGJ_2_ on LPS-induced IL-8/CXCL8 expression was mediated through PPARg in SHR VSMCs. 15d-PGJ_2_ and other cyclopentenone prostaglandin, such as prostaglandin A_1_ (PGA_1_) are known to exert effects on cytokine genes through PPARγ-dependent and PPARγ-independent mechanisms (25,26,28,29,31,32). In our previous studies (25,26), the upregulatory effect of 15d-PGJ_2_ on LPS-induced MIP-2/CXCL2 and KC/CXCL1 gene expression was found to not mediated by the PPARγ pathway, but the effect of PGA_1_ on LPS-induced IL-10 expression was dependent on PPARγ in mouse peritoneal macrophages. In a separate VSMCs study, 15d-PGJ_2_-induced HO-1 expression was shown to be independent of PPARγ (19).

In the EMSA, an increase in NF-κB activity in SHR VSMCs treated with 15d-PGJ_2_/LPS relative to VSMCs treated with LPS alone was detected, and the expression of 15d-PGJ_2_/LPS-induced IL-8/CXCL8 mRNA was decreased by the presence of Bay11-7082. These results indicate that the upregulatory effect of 15d-PGJ_2_ in SHR VSMCs is dependent on NF-κB activation. It is widely accepted that 15d-PGJ_2_ exerts its effects on inflammatory mediated-genes in cells by either inhibiting or activating NF-κB signaling (24,26,33-35). The anti-inflammatory activity of 15d-PGJ_2_ has been shown to be mediated mainly through the inhibition of NF-κB activation (33,34), but 15d-PGJ_2_ has also been shown to upregulate IL-8/CXCL8 and MIP-2/CXCL2 expression through NF-κB activation (24,26).

Among the MAPK signaling pathways, the ERK pathway is known to be associated with the stimulatory activity of 15d-PGJ_2_ on the expression of some cytokine genes (23,24,36). 15d-PGJ_2_ induces the rapid activation of the ERK pathway in VSMCs (37). We also detected an increase in ERK phosphorylation in SHR VSMCs treated with 15d-PGJ_2_/LPS. Up-regulation of LPS-induced IL-8/CXCL8 expression by 15d-PGJ_2_ was mediated through the ERK signaling pathway in SHR VSMCs. Inhibition or activation by 15d-PGJ_2_ on p38 MAP kinase appears to be target gene-, cell type- and stimulation condition-dependent (19,38,39). Activation of the p38 MAP kinase has been shown to be involved in 15d-PGJ_2_-induced HO-1 expression (19) and in IL-1β-induced IL-8/CXCL8 gene expression in human VSMCs (38). However, 15d-PGJ_2_ is known to inhibit IL-1-induced p38 MAP kinase expression in human astrocytes (39). In this study, blocking p38 phosphorylation increased 15d-PGJ_2_/LPS-induced IL-8/CXCL8 expression in SHR VSMCs. 15d-PGJ_2_ inhibited p38 phosphorylation in SHR VSMCs treated with LPS. Furthermore, while the expression of LPS-induced IL-8/CXCL8 mRNA was abolished in SHR VSMCs that were transfected with p38 siRNA, 15d-PGJ_2_/LPS-induced IL-8/CXCL8 expression increased. Therefore, LPS-induced IL-8/CXCL8 mRNA expression appears to be related to p38 activation, but 15d-PGJ_2_ itself induces IL-8/CXCL8 mRNA expression without p38 activation. These combined results indicate that the upregulatory effect of 15d-PGJ_2_ on LPS-induced IL-8/CXCL8 expression is related to p38 inactivation. Although the upregulatory effect of 15d-PGJ_2_ on LPS-induced IL-8/CXCL8 expression is mediated by the ERK pathway, an unknown mechanism via p38 inactivation may play an important role in 15d-PGJ_2_/LPS-induced IL-8/CXCL8 expression in SHR VSMCs.

NAD(P)H oxidase is a known major source of reactive oxygen species (ROS). ROS are not only harmful cellular metabolites but are also essential molecules in cell signaling and regulation (40). Excessive ROS generation by NAD(P)H oxidase is known to play an important role in the pathogenesis of hypertensive vascular injury and has also been implicated in the pathogenesis of hypertension (1,41-44). Cruzado et al. (41) demonstrated that ROS generation was enhanced in SHR VSMCs during the development of hypertension. Therefore, we examined the effect of NAD(P)H oxidase activity on IL-8/CXCL8 expression in SHR VSMCs treated with 15d-PGJ_2_/LPS. An inhibitor of the flavin-containing oxidases, DPI, remarkably decreased the expression of 15d-PGJ_2_/LPS-induced IL-8/CXCL8 mRNA. Although a significant production of ROS by 15d-PGJ_2_ has been reported in Sprague-Dawley rat VSMCs (19) and ROS generation by LPS alone was detected in WKY VSMCs (data not shown), ROS generation did not increase in SHR VSMCs after treatment with 15d-PGJ_2_ or LPS alone. However, 15d-PGJ_2_/LPS did increase ROS generation in SHR VSMCs. These results suggest that the upregulatory effect of 15d-PGJ_2_ on LPS-induced IL-8/CXCL8 expression in SHR VSMCs may be related to NAD(P)H oxidase activity.

In conclusion, this is the first study to report on the upregulatory effect of 15d-PGJ_2_ on LPS-induced IL-8/CXCL8 gene expression in SHR VSMCs and the inhibitory effect in WKY VSMCs. In addition, we showed that the upregulatory effect of 15d-PGJ_2_ in SHR VSMCs is mediated through PPARγ pathway, NF-κB and ERK activation, and p38 inactivation may play an important role in 15d-PGJ_2_/LPS-induced IL-8/CXCL8 expression. These results provide new insight into the potential diverse effects of 15d-PGJ_2_ on hypertensive vascular smooth muscle cells.
